# Chromosome-level genome assembly of *Aquilaria yunnanensis*

**DOI:** 10.1038/s41597-024-03635-z

**Published:** 2024-07-17

**Authors:** Meifei Li, Yingmin Zhang, Yi Wang, Yue Yin, Meijun Zhou, Yonghong Zhang

**Affiliations:** 1 School of Life Sciences, Yunnan Normal University, Kunming, 650500 China; 2 College of Chinese Material Medica, Yunnan University of Chinese Medicine, Kunming, 650500 China; 3 Engineering Research Center of Sustainable Development and Utilization of Biomass Energy, Ministry of Education, Kunming, 650500 China

**Keywords:** Genome, Phylogenetics

## Abstract

*Aquilaria yunnanensis* is an endangered agarwood-producing tree currently listed on the IUCN Red List of Threatened Species. The agarwood it produces has important medicinal and economic value, but its population has sharply declined due to human destruction and habitat reduction. Therefore, obtaining genomic information on *A. yunnanensis* is beneficial for its protection work. We assembled a chromosome-level reference genome of *A. yunnanensis* by using BGI short reads, PacBio HiFi long reads, coupled with Hi-C technology. The final genome assembly of *A. yunnanensis* is 847.04 Mb, with N50 size of 99.68 Mb, in which 805.49 Mb of the bases were anchored on eight pseudo-chromosomes. Two gapless pseudo-chromosomes were detected in the assembly. A total of 27,955 protein-coding genes as well as 74.65% repetitive elements were annotated. These findings may provide valuable resources in conservation, functional genomics, and molecular breeding of *A. yunnanensis*, as well as the molecular phylogenetics and evolutionary patterns in *Aquilaria*.

## Background & Summary

The genus *Aquilaria* of Thymelaeaceae, which is consisted of 21 accepted species so far, is native to Indomalesia region. The members of *Aquilaria* are known to be the primary source of the fragrant non-wood product - agarwood, which is sold as a valuable ingredient for the making of incense, perfume, and traditional medicine^1^. However, the high demand for natural agarwood is ever increasing; yet, natural agarwood is rare to be obtained in the wild. The collection of agarwood from the trees is considered a destructive act, and the over-harvesting and indiscriminate felling of these trees have endangered the survival of the species in the wild^2,3^. At present, all the species of *Aquilaria* has been listed in the Convention on International Trade in Endangered Species of Wild Fauna and Flora (CITES) under the category Appendix II^4^.

**Fig. 1:**
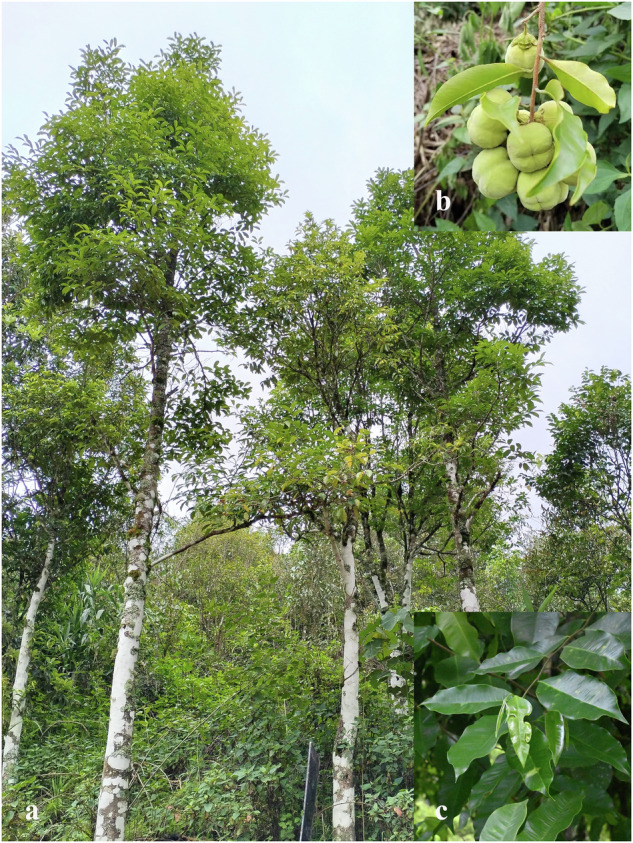
*Aquilaria yunnanensis* S. C. Huang. (**a**) habitat and individuals, (**b**) fruit, and (**c**) twigs and leaves.

*Aquilaria yunnanensis* S. C. Huang is a precious agarwood-producing tree species native to the Yunnan Province of China^5^ (Fig. 1). Based on the latest assessment, *A. yunnanensis* is categorized as “Vulnerable” under the criteria B1ab(i) by the International Union for Conservation of Nature (IUCN) Red List of Threatened Species^6^. Due to the decline of suitable habitat for survival, the species is now experiencing a narrow distribution and diminishing populations^7^. Unlike its congener, *Aquilaria sinensis*, *A. yunnanensis* is only confined to 10 locations in Yunnan, while *A. sinensis* is widely distributed in at least six provinces of China. Despite a new population of *A. yunnanensis* was recently discovered in the northern region of Vietnam, there were only less than 10 mature individuals recorded in that area^8^. Such phenomenon has somewhat gained the attention of local researcher to conserve its population. Although sufficient genetic information of this tree could lay out a foundation to strategizing the conservation effort of this vulnerable species, when compared to its congener, *A. sinensis*, the genomic information for *A. yunnanensis* is still very limited at present, however.

In order to provide genome-scale insights into this vulnerable species, we assembled the first high-quality chromosome-level reference genome sequence for *A. yunnanensis* using BGI short reads and PacBio long reads, coupled with the Hi-C technology. We determined that the primary genome assembly was approximately 846.95 Mb and had a contig N50 of 87.04 Mb. Using Hi-C data, we determined that 805.49 Mb (95.10%) of the assembled bases were assigned to eight pseudo-chromosomes. The final genome assembly of *A. yunnanensis* was 847.04 Mb. A total of 74.65% of the genome was occupied by repetitive sequences, of which the long terminal repeats (LTR) were predominant (48.28% of the whole genome). Gene prediction identified 27,955 protein-coding genes, of which 22,096 of them were presumably functional.

The availability of the genome information provides a valuable foundation not only for the studies of phylogenetic relationship, genetic diversity, functional genomics, and genomics-assisted breeding of *A. yunnanensis*, at the same time, also facilitates the comparative genetics and genomic research of *Aquilaria*.

## Methods

### Sample collection, library construction and sequencing

Total genomic DNA of *A. yunnanensis* were extracted from fresh leaves collected from Bubang village of Mengla County, Yunnan Province (21°35′59″N, 101°34′47″E; Fig. 1) using modified CTAB method^9^. The DNBSEQ-T7 library utilized 1 μg of DNA per sample as the input material, and the sequencing libraries were constructed using the VAHTS Universal DNA Library Prep Kit for MGI (Vazyme, Nanjing, China) according to the manufacturer’s protocol. The quantification and size of the library were measured using Qubit 3.0 Fluorometer (Life Technologies, Carlsbad, CA, USA) and Bioanalyzer 2100 system (Agilent Technologies, CA, USA)^10^. The library was subjected to DNA nanoball (DNB) generation and was subsequently sequenced on a DNBSEQ-T7 (BGI, Shenzhen, China) sequencer with DNBSEQ-T7RS Sequencing Reagent in paired-end 150 bp mode^11^. In total, 441 million reads were generated, amounting to 132.30 Gb of raw sequence data.(Supplementary Table 1).

**Fig. 2:**
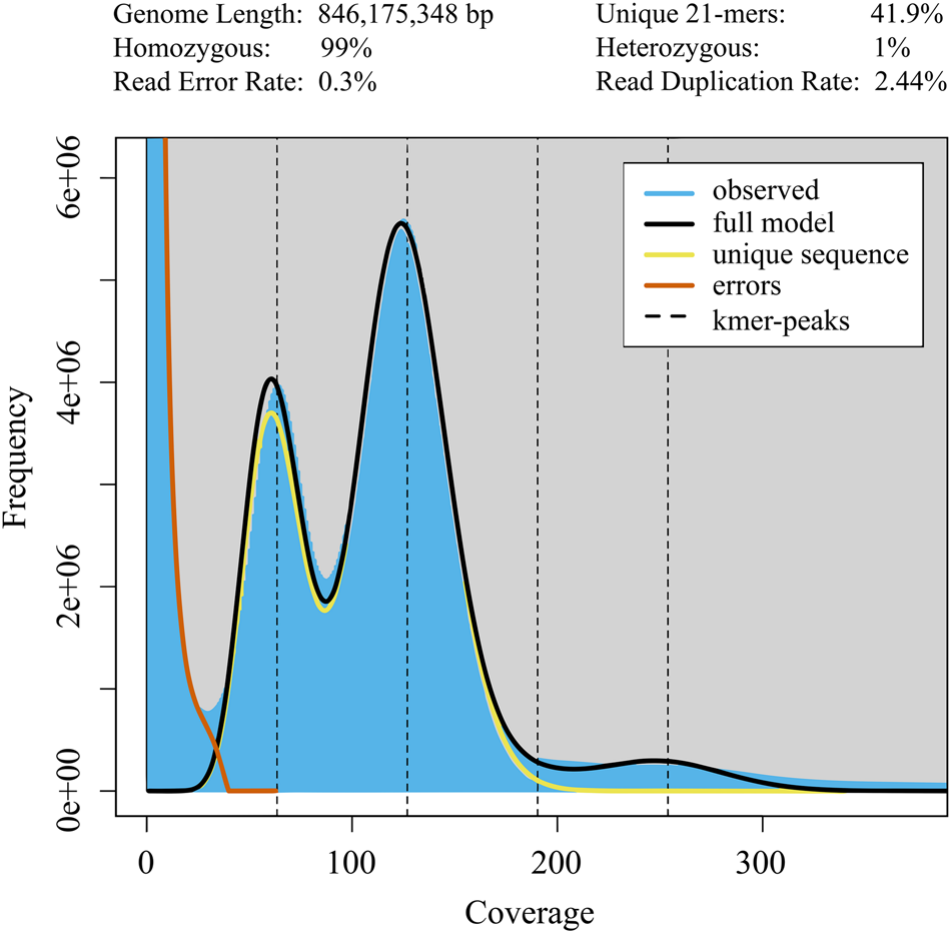
K-mer analysis (k = 21) for the genome size estimation of *A. yunnanensis*. The blue area denotes the observed 21-mer frequency distribution. The fitted model is indicated as a black line. The unique and putative error k-mer distributions are shown as yellow and red, respectively.

To construct the PacBio HiFi library, the DNA template was sheared to an average size of 15 kb with g-TUBE (Covaris, Inc., MA, USA), and the target DNA fragments were recovered using BluePippin size selection System (Sage Science, Inc, MA, USA). The SMRTbell library was constructed using the SMRTbell Express Template Prep kit 2.0 (Pacific Biosciences, California, USA), according to the manufacturer’s instructions. The SMRTbell library was introduced to the PacBio Sequel II platform (Pacific Biosciences, Menlo Park, USA) for sequencing, and the consensus reads (HiFi reads) were generated utilizing the Circular Consensus Sequencing (CCS) software (https://github.com/pacificbiosciences/unanimity) with the parameter ‘-minPasses 3’^12^. Approximately 31.75 Gb data were obtained, in which the average length was 15,365 bp and the N50 length was 15,576 bp, after removing the adaptors in polymerase reads (Table 1).

**Table 1:** Sequencing data of *A. yunnanensis* genome based on four different types of libraries.

Library type	Type	Clean data (Gb)	Read N50/length (bp)^a^	Coverage (×)	SRA accession number
**DNBSEQ-T7**	PE	131.73	150	149.55	SRR25783949
**PacBio HiFi**	CCS	31.75	15,576	37.48	SRR25784776
**Hi-C**	PE	20.56	150	24.28	SRR25822242
**RNA**	Subreads	73.16	1,194	—	SRR25817263

^a^The value indicated under the PacBio HiFi and RNA is referred to the N50 length, while the value indicated for other library types is the read length.

Fresh leaf tissue of *A. yunnanensis* was used to construct a library for the Hi-C analysis. The fresh tissue was cross-linked with formaldehyde, and cells were lysed using Nuclear Isolation Buffer lysis solution. Then chromatin DNA were digested with restriction endonuclease (MboI), and sticky ends were formed at the cleavage sites. Sticky ends were biotinylated and proximity-ligated to form chimeric junctions that were enriched. Finally, the DNA samples were purified, impurities removed, and randomly interrupted into fragments of 300–500 bp size for library construction. Purified DNA was further blunt-end repaired, A-tailed and adaptor-added, prior to purification through biotin-streptavidin-mediated pull-down and PCR amplification. The Hi-C libraries were quantified and sequenced on the Illumina Nova-seq platform (Illumina, San Diego, CA, USA), which generated a total of 258,748,211 pairs of reads. The 77.62 Gb raw data had a coverage of 91.64× of the genome.

### RNA preparation and sequencing

The RNA samples were extracted from roots, stems and leaves tissues using the standard Trizol reagent (Invitrogen, CA, USA) and equally mixed for sequencing. RNA purity and integrity was monitored with NanoDrop 2000 spectrophotometer (NanoDrop Technologies, Wilmington, DE, USA)and an Agilent 2100 Bioanalyzer (Agilent Technologies, CA, USA). RNA contamination was assessed using 1.5% agarose gel electrophoresis. The full-length cDNA was synthesized using a Clontech SMARTer PCR cDNA Synthesis Kit (Takara Biotechnology, China). Then, the SMRTbell libraries were constructed using the Pacific Biosciences SMRTbell template prep kit (Pacific Biosciences, USA). The libraries’ quantification and size were measured using Qubit 3.0 Fluorometer (Life Technologies, Carlsbad, CA, USA) and Bioanalyzer 2100 system (Agilent Technologies, CA, USA). Transcriptome sequencing was conducted using Iso-seq under the CCS model. Subsequently, SMRTbell sequencing was performed on a PacBio Sequel II platform by Frasergen Bioinformatics Co., Ltd. (Wuhan, China). After removing adaptors in polymerase reads, a total of 73.16 Gb subreads were obtained with an average length of 1,122 bp and an N50 length of 1,194 bp.

### Genome size and heterozygosity estimation

The generated short reads from the DNBSEQ-T7 platform were subjected to qualitative filtering using SOAPnuke v2.1.6^13^ based on the following approaches: the adaptors were removed from the sequencing reads; read pairs were excluded if either end had an average quality of <20; ends of reads were trimmed when the average quality was <20 in the 5-bp sliding window analysis; then removed the read pairs with either ends shorter than 75 bp. A clean data of 131.73 Gb was obtained for assessing the characteristics of the genome (Table 1). The 21-mer frequency distribution of sequencing reads from the short reads was generated using Jellyfish v2.1.4^14^. Using the software GenomeScope v2.0^15^, the genome size was estimated to be about 846.18 Mb, and the proportion of repeat sequences and heterozygosity rate of the genome were determined to be approximately 58.1% and 1.0% (Fig. 2), respectively.

### *De novo* genome assembly

The PacBio HiFi reads were used for *de novo* assembly using hifiasm v.0.14-r312^16^ with default parameters. Gfatools (https://github.com/lh3/gfatools) was used to convert the sequence graphs from the GFA format into FASTA format. The primary assembly was corrected using short reads from the DNBSEQ-T7 library, and the correction process was completed using Pilon v1.23^17^. As a result, the *A. yunnanensis* genome assembly had a total length of about 846.95 Mb, which contained 575 contigs; while the contig N50 was 87.04 Mb (Table 2).

**Table 2:** Information of *A. yunnanensis* genome assembly based on the Hifiasm-derived contigs and Hi-C scaffolded assembly.

Key	Hifiasm-derived contigs	Hi-C scaffolded assembly
Total length	846,952,581	847,035,581
Contig/scaffold number	575	415
Contig/scaffold N50	87,043,176	99,675,900
Average contig/scaffold length (bp)	1,472,961	2,041,049
Largest contig/scaffold length (bp)	122,106,131	119,538,367
GC content (%)	38.2	38.2

**Fig. 3:**
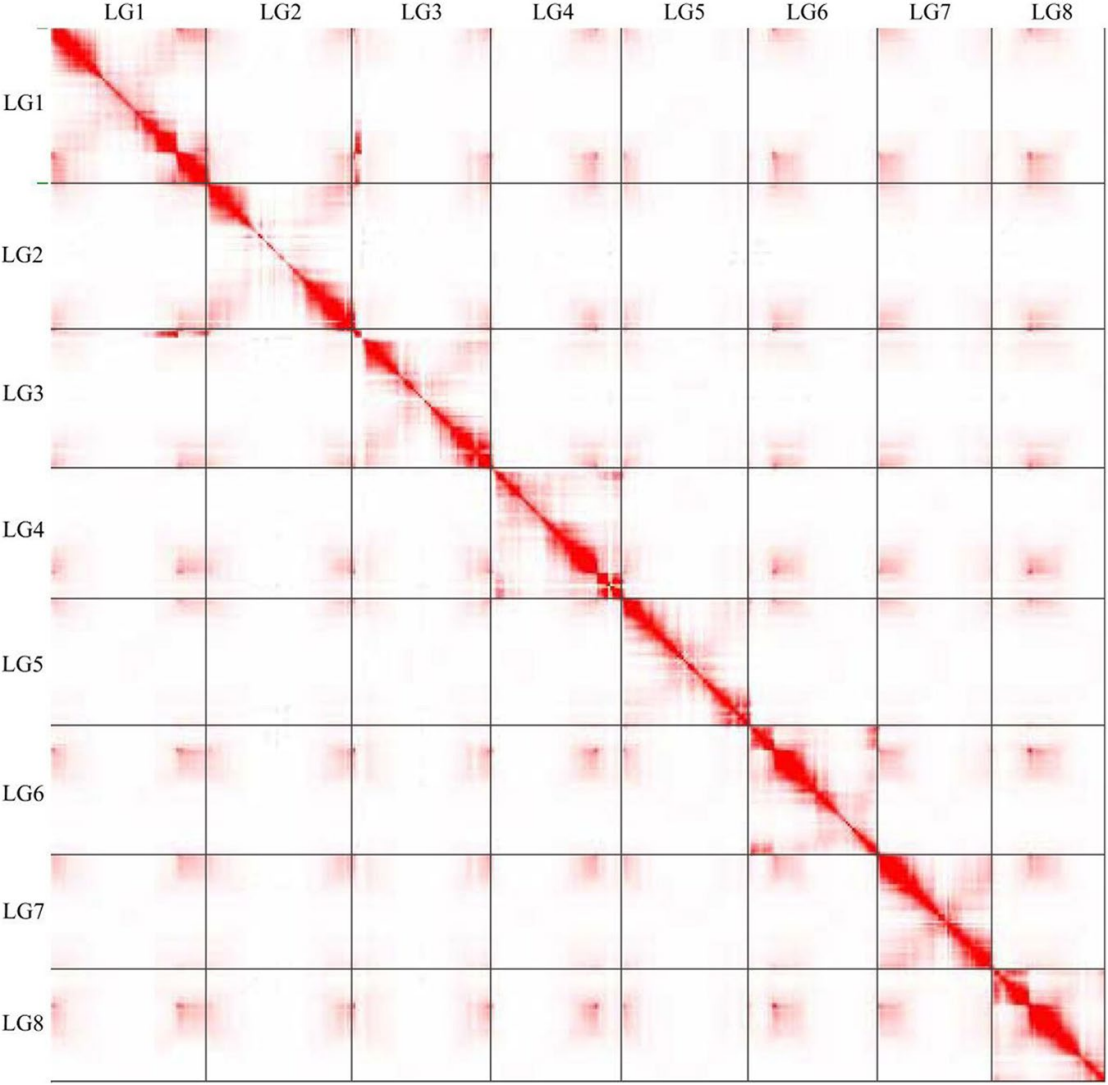
Hi-C interaction heatmap at chromosome-level of *A. yunnanensis*. The heatmap indicates that the intra-chromosome interactions (blocks on the diagonal line) are stronger compared to the inter-chromosome interactions.

**Fig. 4:**
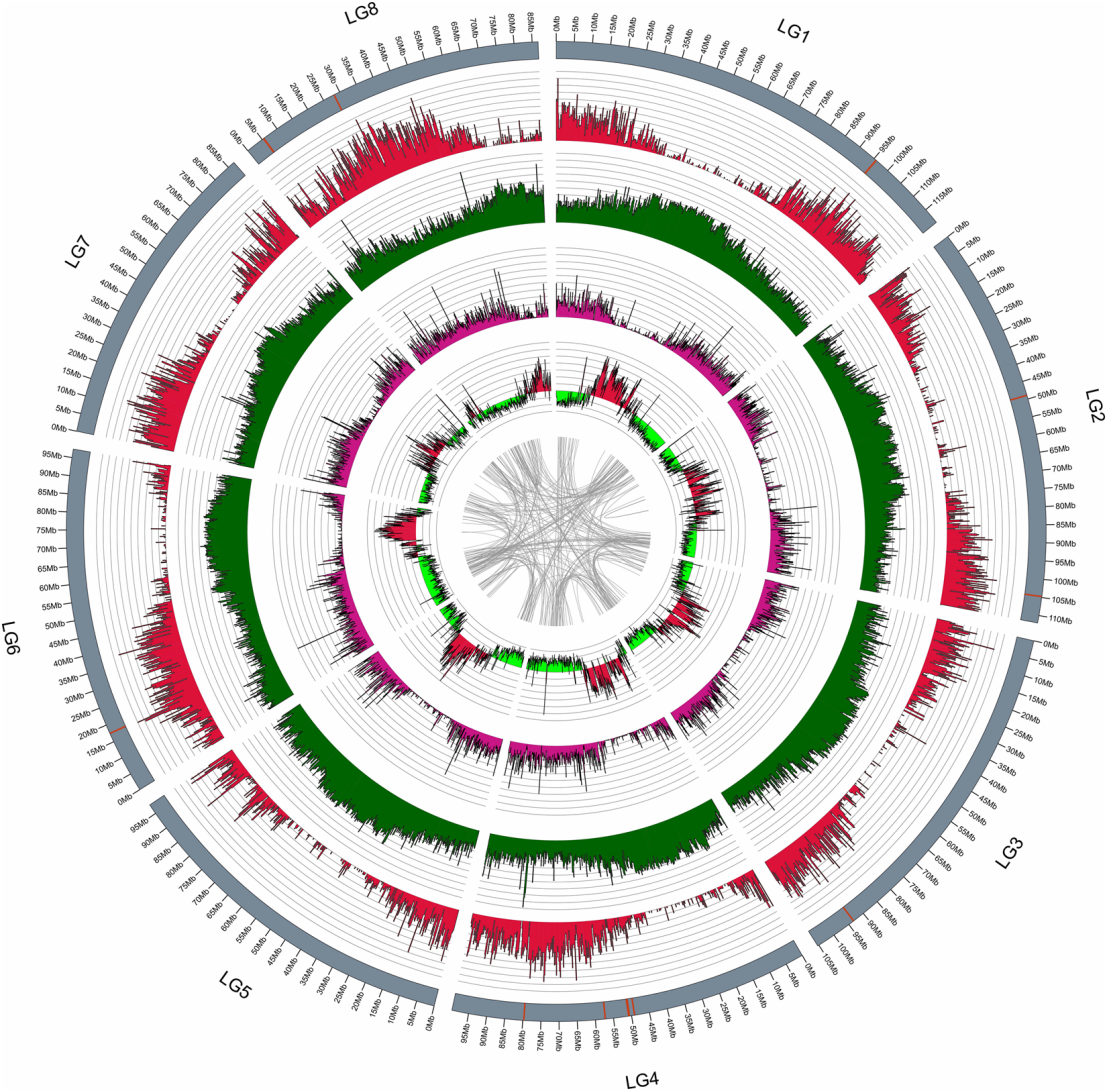
A circos plot of eight chromosomes of *A. yunnanensis* genome. The tracks from outside to inside represent: gene density, transposon density, repeat sequence density, GC content, and collinear blocks. The red vertical lines on the outside track indicate the gaps.

The raw Hi-C data were primarily filtered using Fastp^18^, followed by mapping the filtered Hi-C data to the *A. yunnanensis* genome using Bowtie2 v2.3.2^19^ with the default parameters. An iterative mapping strategy was employed, retaining only read pairs with uniquely mapped both ends for the subsequent analysis, to increase the ratio of interactive Hi-C reads. Self-ligation, non-ligation, and other invalid reads, including StartNearRsite, PCR amplification, random break, LargeSmallFragments, and ExtremeFragments, were filtered out by HiCUP^20^. A total of 20.56 Gb clean data were retained (Table 1). The order and orientation of the clustered contigs were arranged with D-DNA v180922 pipeline^21^. The construction of the chromosome was manually carried out using the Juicebox tool package v1.22.01^22^. A total of 575 contigs were used to construct scaffolds with Hi-C data, which generated 415 scaffolds consequently (Table 2). The scaffolds were anchored on eight pseudo-chromosomes (Fig. 3). Among them, six pseudo-chromosomes contained a total of 16 gaps, each with a length of 500 bp, while the other two pseudo-chromosomes were gapless (Fig. 4, Supplementary Table 2). The Hi-C-assisted chromosome-length scaffolds yielded a final size of 805.49 Mb accounting for the 95.10% draft genome, ranging from 86.64 Mb to 119.54 Mb in length (Table 3).

**Table 3:** Information of the *A. yunnanensis* genome at chromosomal-level.

Chromosome ID	Chromosome size (bp)	Contig number	Contig size (bp)	GC content (%)
LG1	119,538,367	4	119,536,867	38.07
LG2	112,076,725	3	112,075,725	37.97
LG3	105,361,115	2	105,360,615	38.00
LG4	99,675,900	6	99,673,400	38.21
LG5	97,814,526	1	97,814,526	38.29
LG6	97,343,320	2	97,342,820	38.54
LG7	87,043,176	1	87,043,176	37.02
LG8	86,640,963	5	86,638,963	37.03
ChrAll	805,494,092	24	805486092	37.92

### Repeat annotation

The *de novo*- and homology-based approaches were used to identify the repeat sequences in the *A. yunnanensis* genome assembly. RepeatModeler v2.0.4^23^ was used to construct a *de novo* repeat library, which employed the results from RECON v1.0.8^24^ and RepeatScout v1.0.6^25^. For the homology-based approach, repeats was identified using RepeatMasker v4.1.5^26^, integrating both the Repbase library (http://www.girinst.org/repbase/) and the *de novo* repeat library, to detect known transposable elements (TEs) within the genome assembly. The results indicated a total of 632.35 Mb repetitive sequences identified, representing 74.65% of the *A. yunnanensis* genome assembly. The LTR elements, accounting for 48.28% of the whole genome, were the most abundant. For other classes, the DNA transposons, long interspersed nuclear elements (LINE), and short interspersed nuclear elements (SINE) had accounted for 6.09%, 1.65%, and 0.01% of the whole genome, respectively (Table 4)

**Table 4:** Information on the repeat annotation in the *A. yunnanensis* genome assembly.

Type			Number of elements	Sequence length(bp)	Percentage of genome (%)
Retroelements	LTR elements	Gypsy/DIRS1	183,375	323,373,188	38.18
		Ty1/Copia	56,266	53,943,205	6.37
		Retroviral	1,966	135,655	0.02
		BEL/Pao	1,794	663,112	0.08
	Non-LTR elements	LINEs	28,142	13,971,685	1.65
		SINEs	1,312	117,378	0.01
		Penelope	641	163,742	0.02
	Total of retroelements		321,230	423,020,254	49.94
DNA transposons			119,527	51,582,681	6.09
Rolling-circles			6,767	5,761,739	0.68
Unclassified			423,644	131,016,515	15.47
Total interspersed repeats				605,783,192	71.52
Small RNA			6181	15,840,470	1.87
Satellites			2021	339,821	0.04
Simple repeats			93,120	3,810,942	0.45
Low complexity			17,451	863,140	0.10
Total			1,099,317	632,352,644	74.65

### Gene prediction and functional annotation of the genome

For annotation of the protein-coding genes, we employed a method integrating transcriptome-based, *ab initio*, and homologue-based strategies to identify the gene models using Maker v3.01^27^. For the transcriptome-based gene prediction, we used the CCS, lima (https://github.com/pacificbiosciences/barcoding/) and IsoSeq (https://github.com/pacificBiosciences/pbbioconda) pipelines to obtain the transcript sequences. Error correction was carried out on the raw sequencing data using the CCS v6.4.0; while the adaptor sequences were filtered using lima v2.7.1. Further sequence filtering and clustering were conducted using IsoSeq v4.0.0 to produce accurate full-length transcript sequences, which were used as input data for the Maker software. The *ab initio* gene prediction was conducted using Augustus v3.4.0^28^; while the proteins sequences from *Aquilaria sinnsis*^29^, *Arabidopsis thaliana*^30^, *Gossypium hirsutum*^31^, *Stellera chamaejasme*^32^ and *Theobroma cacao*^33^ were aligned with the genome of *A. yunnanensis* using TBLASTN^34^. The homologous genes were identified using Exonerate v2.2.2^35^. As the gene prediction via Maker is based on the transcript sequences, the gene structure models generated by Maker were used as input to train the species-specific model files in Augustus. The gene model prediction was carried out another round using Maker, but with an automatic annotation integration of data, including the transcript evidence, protein evidence, and Augustus gene predictions, into a consensus annotation based on their evidence-based weights. After filtering off genes with protein-encoding sequence that were shorter than 50 amino acids, as well as genes that contained internal stop codons, and illegal start or stop codons, the gene prediction identified a total of 27,955 protein-coding genes being annotated in the *A. yunnanensis* genome.

Functional annotation was performed using eggNOG-mapper v2.1.7^36^ with reference to the eggNOG orthology database and sequence searches were carried out using DIAMOND^37^. Additionally, protein annotation was conducted using eggNOG-mapper by referring to the Gene Ontology (GO) terms and Kyoto Encyclopedia of Genes and Genomes (KEGG) pathways. As a result, a total of 22,096 genes that are presumably functional were annotated, while as much as 12,560 and 7,259 genes were assigned to a specific GO term and a KEGG pathway, respectively.

## Data Records

The BGI short reads, PacBio HiFi long reads, Hi-C reads, and RNA-Seq data were deposited in the National Center for Biotechnology Information (NCBI) Sequence Read Archive (SRA) database with the accession number SRP457418^38^ under BioProject accession number PRJNA1008918^39^. The genome assembly had been deposited in DDBJ/ENA/GenBank under the accession number JBDJPA000000000^40^. The genome assembly and annotation files were submitted to Figshare^41^.

## Technical Validation

### Accuracy assessment of genome assembly

The software BWA v0.7.17-r1188^42^ was used to align the short reads of DNBSEQ-T7 library of *A. yunnanensis* to the assembled genome, achieving 99.51% of mapping rate, with coverage of 99.9%. Merqury v1.3^43^ was used to assess the consensus quality value (QV) of the *A. yunnanensis* genome assembly. The QVs were 65.60 and 46.38 estimated with HiFi and BGI k-mers, respectively, indicating high accuracy of the genome assembly (Supplementary Figure 1).

### Integrity assessment of genome assembly

**Fig. 5:**
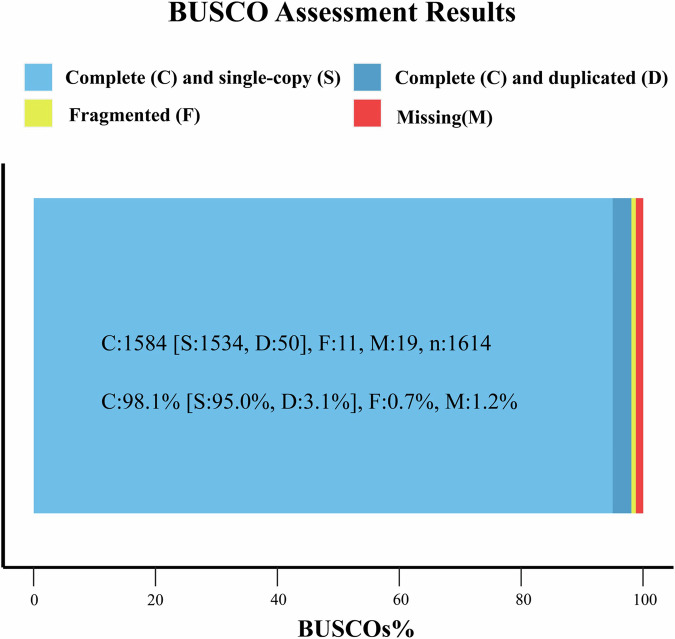
BUSCO scores of the genome assembly of *A. yunnanensis*. C: the number of complete genes, S: the number of complete and single-copy genes, D: the number of complete and duplicated genes, F: the number of incomplete genes, M: the number of missing genes.

The integrity of the final genome assembly was assessed by using BUSCO v5.1.2^44^ with the embryophyta_odb10 orthologous database (https://busco-data.ezlab.org/v5/data/lineages/) including 1,614 widely conserved single-copy genes in embryophytes. The BUSCO analysis revealed that 98.1% of the complete genes were retrieved in the genome, with 95.0% being single-copy and 3.1% duplicated. Only 0.7% and 1.2% of BUSCO genes were fragmented and missing, respectively (Fig. 5). LTR_finder v.1.5.10^45^, LTR_harvest v1.06^46^ and LTR_retriver v2.9.0^47^ were employed to assess the LTR Assembly Index (LAI) value of the genome assembly. The obtained LAI value was 22.16, which achieved the gold standard for genome assembly. The above evaluation results indicate that the *A. yunnanensis* genome assembly has high integrity.

### Supplementary information

Supplementary information

## Data Availability

**The software used in the Methods section was executed with default parameters, with the following exceptions:** SOAPnuke v2.1.6, parameters: -lowQual = 20, -nRate = 0.005, -qualRate = 0.5. GenomeScope v2.0, parameters: -k = 21 -m = 10000 3D-DNA v180922, parameters: -s = MboI. RepeatMasker v4.1.5-p1, parameters: -xsmall -gff. CCS v6.4.0, parameters: --min-rq 0.9 -j 60. lima: v2.7.1, parameters: --isoseq -peek-guess. Maker v3.01, parameters, maker_opt.ctl: est2genome = 1 protein2genome = 1 min_protein = 50 run: mpiexec -n 60 maker. eggNOG-mapper v2.1.7, parameters: --ittype proteins -m diamond –cpu 60. BUSCO v5.1.2, parameters: -m = geno, -l = embryophyta_odb10.
