# Supplementary material for: Chromosome-level genome assembly of Aquilaria yunnanensis
Source: Sci Data. 2024 Jul 17;11:790. doi: 10.1038/s41597-024-03635-z (PMC11255207; doi:10.1038/s41597-024-03635-z)
Supplement: Supplementary file 1 — Supplementary information [file 41597_2024_3635_MOESM1_ESM.pdf]

# Supplementary information

Supplementary Table 1 Statistics of genomic of the DNBSEQ-T7 library. ................................. 2

Supplementary Table 2 The position of the gaps on the chromosome ........................................ 2

Supplementary Figure 1 Merqury copy number spectrum plots for the assembly of *Aquilaria yunnanensis* generated with short reads of DNBSEQ-T7 library (a) and PacBio (b) reads............ 3

Supplementary Table 1 Statistics of genomic of the DNBSEQ-T7 library.

| **Sample** | **Length (bp)** | **Q20 (%)** | **Q30 (%)** | **GC Content (%)** | **Total Reads** | **Total Bases** |
|---|---|---|---|---|---|---|
| *A. yunnanensis* | 150; 150 | 97.77; 95.27 | 92.84; 86.43 | 38.56; 38.69 | 440,999,615 | 132,299,884,500 |

Supplementary Table 2 The position of the gaps on the chromosome

| **Gap ID** | **Chrmosome ID** | **Start Position** | **End Position** |
|---|---|---|---|
| 1 | LG1 | 95713851 | 95714351 |
| 2 | LG1 | 95734372 | 95734872 |
| 3 | LG1 | 95788413 | 95788913 |
| 4 | LG2 | 48973893 | 48974393 |
| 5 | LG2 | 104716169 | 104716669 |
| 6 | LG3 | 93356677 | 93357177 |
| 7 | LG4 | 49143504 | 49144004 |
| 8 | LG4 | 50557105 | 50557605 |
| 9 | LG4 | 50792625 | 50793125 |
| 10 | LG4 | 57258751 | 57259251 |
| 11 | LG4 | 79707187 | 79707687 |
| 12 | LG6 | 17519041 | 17519541 |
| 13 | LG8 | 5439944 | 5440444 |
| 14 | LG8 | 5515826 | 5516326 |
| 15 | LG8 | 5543453 | 5543953 |
| 16 | LG8 | 28266992 | 28267492 |

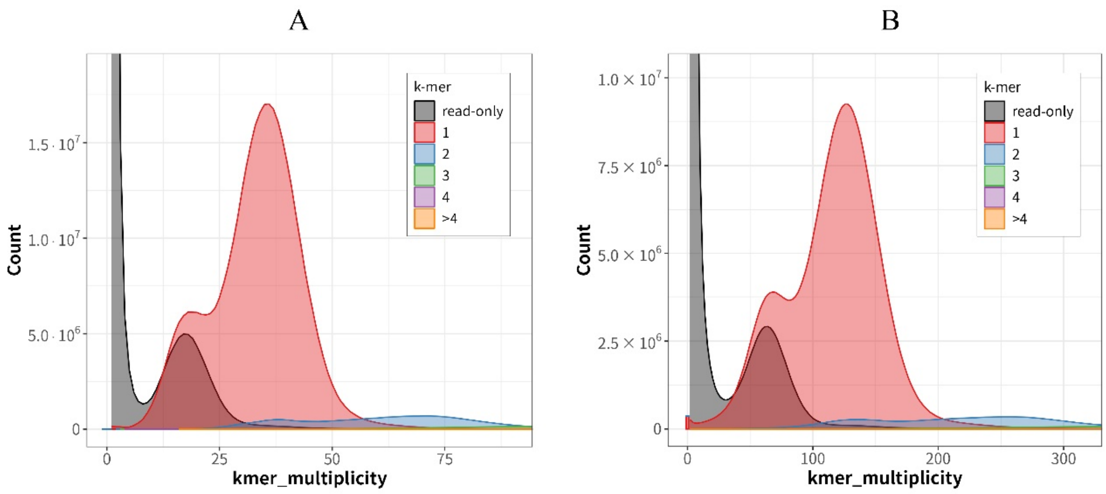


Supplementary Figure 1 Merqury copy number spectrum plots for the assembly of *Aquilaria yunnanensis* generated with short reads of DNBSEQ-T7 library (a) and PacBio (b) reads.
